# Identification of a Novel Tumor Inflammation Signature for Risk Stratification, Prognosis Prediction, and Immune Status in Colorectal Cancer

**DOI:** 10.1155/2022/3465391

**Published:** 2022-07-16

**Authors:** Yayun Li, Zhengcai Qiu, Qipeng Wang, Shangshang Li, Qiong Zhang, Jing Han

**Affiliations:** ^1^Shuyang Hospital of TCM, Jiangsu Province, China; ^2^First Affiliated Hospital of Xuzhou Medical University, China

## Abstract

**Background:**

Inflammation and immune cell dysfunction have been widely known as an essential role in the tumorigenesis of colorectal cancer (CRC). Yet, the role of tumor inflammation signature (TIS) associated with CRC prognosis, immune infiltration, and drug resistance remained unknown.

**Method:**

The transcriptome sequencing data, as well as clinical data of CRC from the public dataset, were acquired for further investigation. Inflammation-related gene expression patterns were obtained and analyzed. Bioinformatics methods were used to build a prognostic TIS, and its prediction accuracy was verified by using ROC curve analyses. The independent prognostic factors in CRC were identified through multivariable Cox regression analysis. In addition, the specific features of the immunological landscape between low- and high-risk CRC cohorts were analyzed.

**Results:**

We firstly screened the differentially expressed inflammation-related genes in CRC and constructed a prognostic TIS. We further classified CRC patients into high or low TIS score groups based on the optimal cutoff of prognostic TIS, and patients with high-risk scores had shorter overall survival (OS) than those in the low-risk cohort. The diagnostic accuracy of TIS was evaluated and confirmed with ROC analysis. The result of the univariate and multivariate analysis found that TIS was directly and independently linked to OS of CRC. Otherwise, an optimal nomogram model based on TIS exhibited a better prognostic accuracy in OS. Finally, the immunological status and immune cell infiltration were observed different in the two-risk cohorts.

**Conclusion:**

In summary, the risk model of the TIS in CRC was found to be useful for predicting patient prognosis and guiding individual treatment. This risk signature could also serve as potential biomarkers and immunotherapeutic targets and indicate immunotherapy response for patients with CRC.

## 1. Introduction

Colorectal cancer (CRC) is the most common primary digestive system tumor and ranks third among all cancers in incidence and mortality worldwide. The incidence of early-onset CRC has increased in many countries [1]. The main treatment for CRC is curative surgery combined with adjuvant chemotherapy. However, the OS of CRC patients is still low as those diagnosed at the advanced stage [2]. The TNM staging (tumor, lymph node, and metastasis) system is widely used in the clinical setting for prognostic prediction of CRC [3]; however, when TNM staging was used alone in clinical practices, it is not enough in predicting survival and making treatment options for CRC patients.

The role of chronic inflammation is pivotal in the initiation and progression of many diseases, including cancer [4]. Chronic inflammation, together with genetic and epigenetic changes, has been shown to lead to the initiation of CRC. The incidence of CRC in patients with inflammatory bowel disease is higher, which is more likely to be caused by long-standing inflammatory disease of the colon [5]. Recent findings have shown that inflammatory pathways not only are important in the pathogenesis of CRC but are also involved in the development of CRC [6]. Growing evidence supports that various proinflammatory pathways promote tumorigenesis by inducing the production of inflammatory mediators. On the one hand, inflammation promotes tumor progression by contributing to malignant conversion, invasion, and metastasis and also makes tumor cells escape from immune surveillance and results in tolerance to chemotherapeutic drugs or immunotherapy [7].

In this study, we aimed to develop a tumor inflammation signature (TIS) to explore the role of inflammation-related genes in CRC. A better understanding of the TIS of CRC may provide some promising targets for prognosis and therapy.

## 2. Materials and Methods

### 2.1. Data Acquisition and Ethics Statement

The clinical features and RNA-seq expression data of patients who had a pathological diagnosis of CRC were collected from TCGA databases (https://portal.gdc.cancer.gov/). Patients with missing survival data will be excluded from the cohorts. In total, 488 CRC patients and 42 normal control patients were enrolled for further study. As TCGA is open publicly available databases, the data collected from the databases was compliant with all applicable laws, regulations, and policies for the protection of human subjects, and all written informed consents were obtained from all subjects involved.

### 2.2. Identification and Construction of the Prognostic TIS Signature

We extracted the inflammation-related genes for bioinformatics from the human gene database (https://http://www.genecards.org/). The “limma” package was carried out to calculate the differentially expressed genes (DEGs) between CRC tissues and nontumor tissues. The prognostic inflammation-related genes were selected through the univariate Cox analysis. Then, candidate inflammation-related genes were selected to construct TIS through the LASSO regression analysis. The risk score was calculated by using the following formula: risk score = sum (expression (each TIS) × corresponding coefficients (each TIS)).

### 2.3. Functional Enrichment Analysis

The potential function and possible molecular mechanisms of TIS in CRC were analyzed by the Gene Set Enrichment Analysis (GSEA) software.

### 2.4. Immune Landscape Analysis

As a specific algorithm in the R package gsva, ssGSEA was performed to estimate immune infiltration levels and immune-related functions. The CIBERSORT and ESTIMATE algorithms were used to analyze the levels of stromal cells and immune cell infiltration. The relationship between risk score and stromal/immune score was calculated by using Pearson's correlation analyses. Two-way ANOVA was used for comparing the differences between the different types of immune infiltration. We adopted the Spearman correlation test to investigate the correlation between the risk score and tumor stemness.

### 2.5. Analysis of the Sensitivity to Potential Drugs

The NCI-60 dataset, an anticancer cell line panel of 9 different histopathological origins, was used to gain new insights into cancer drug response. The CellMiner web application was used to obtain the NCI-60 dataset for further study. The data distribution and comparison between drug sensitivity and prognostic gene expression were analyzed by Pearson's analysis.

### 2.6. Statistical Analysis

LASSO analyses were adopted to screen out prognosis-related TIS. The Kaplan-Meier analysis was used for survival analysis. The ROC curve was put into use to evaluate the predictive accuracy of TIS. The Wilcoxon test was conducted to calculate the association between TIS and clinical variables (gender, age, etiology, and TNM stage). Throughout the text, any statistical test was considered statistically significant with a *p* value < 0.05. The statistical tests were done with the R software using the appropriate packages.

## 3. Results

### 3.1. Data Preparation

Among these patients screened from TCGA, 530 patients were enrolled in this study, 488 patients who were diagnosed with CRC, and 42 normal colon tissues. The baseline transcriptome data as well as the corresponding clinical characteristics of these individuals were publicly collected from TCGA. A database called GeneCards was used to obtain the 200 inflammation-related genes.

### 3.2. Identification of Differentially Expressed TIS

**Figure 1:**
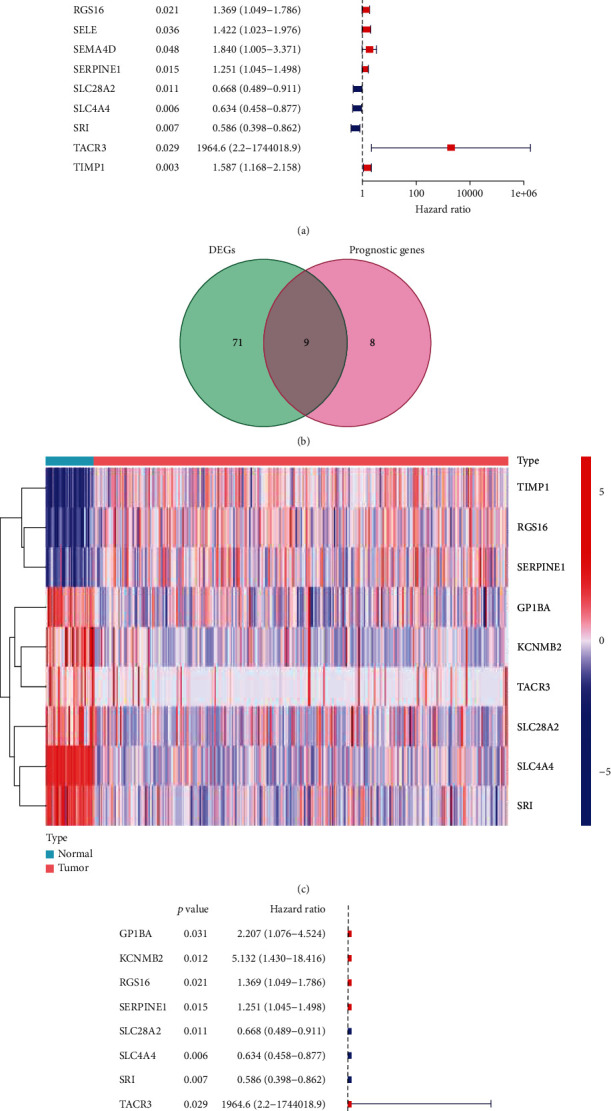
Validation of tumor inflammatory-related genes. (a) Forest plots visualizing considering prognostic tumor inflammatory response-related genes. (b) Prognostic DEGs were identified by the Venn diagram. (c) The heat map of nine prognostic DEGs between CRC tissues and normal tissues. (d) The hazard ratio of multivariate Cox proportional hazard regression analysis for the prognostic DEGs.

**Figure 2:**
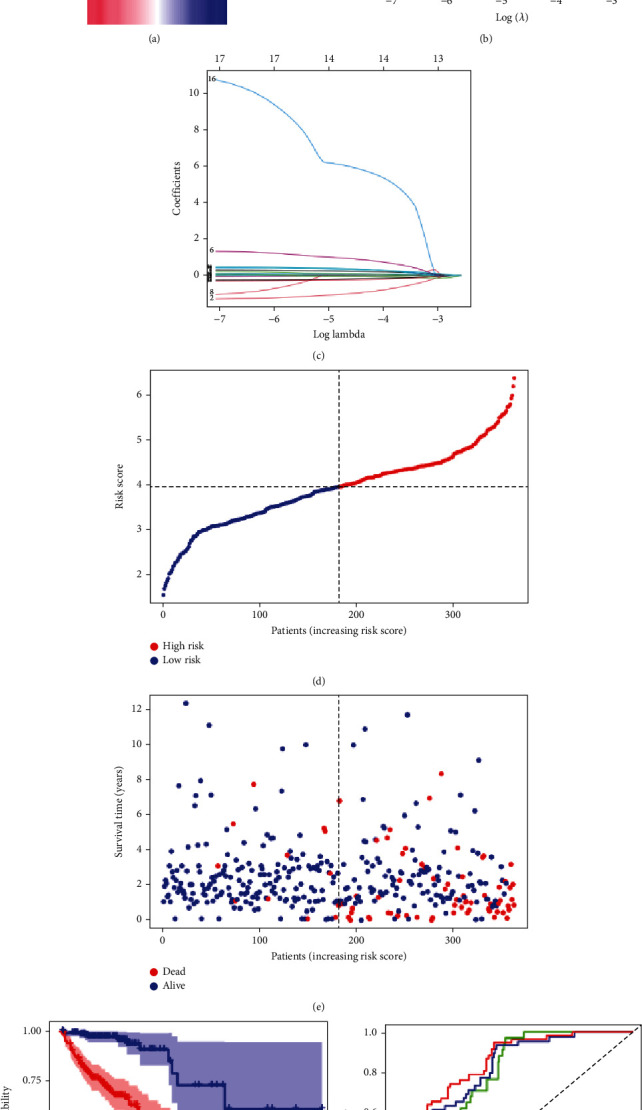
Construction of a prognostic TIS model. (a) The correlation network of candidate genes. (b) LASSO coefficient expression profiles of 14 candidate genes. (c) The optimal values of the penalty parameter were evaluated by the tenfold cross-validation. (d, e) The scatter plot of risk score and survival status of prognostic TIS in CRC. (f) Kaplan-Meier curves of CRC patients' overall survival between the high- or low-risk group. (g) The ROC analyses for 1-year, 2-year, and 3-year survival prediction of TIS.

After analyzing these 200 inflammation-related genes, there were 80 DEGs between the CRC and normal group (Supplement table1). Through the univariate Cox regression analysis, 17 prognosis-related genes were determined in TCGA-CRC cohort (Figure 1(a)). The overlapped 9 genes between differentially expressed genes and prognosis-related inflammation genes were calculated by the Venn diagram (Figure 1(b)). Distinctive gene profiles of each characteristic gene between high- and low-risk subgroups were displayed by a heat map (Figure 1(c)). Multivariate Cox proportional hazard regression analyses indicated that 9 inflammatory response-related genes were independent predictors of OS (Figure 1(d)). We further present the correlation network of prognosis-related inflammation genes (red line in Figure 2(a) represents the positive correlation, and blue line represents the negative correlation).

### 3.3. Construction of the TIS Prognostic Model

We applied LASSO algorithms to identify TIS prognostic features (Figures 2(b) and 2(c)). Eventually, fourteen-gene markers were used to construct the TIS. The risk score = (0.0304 × expression BST2) + (−0.9883 × expression CCL22) + (−0.0487 × expression CCRL2) + (0.1033 × expression CX3CL1) + (0.0163 × expression GABBR1) + (0.9371 × expression GP1BA) + (0.2500 × expression IRF7) + (0.1166 × expression RGS16) + (0.3453 × expression SELE) + (0.3730 × expression SEMA4D) + (−0.1832 × expression SLC28A2) + (−0.2370 × expression SLC4A4) + (5.9975 × expression TACR3) + (0.3425 × expression TIMP1).

The CRC patients were categorized into the high-risk and low-risk groups using the risk score median as the threshold (Figure 2(d)). The distribution of survival status shows that patients with poor prognosis (dead) in the high-risk subgroup were more common compared with the low-risk subgroup (Figure 2(e)). When comparing survival differences in each subgroup by the Kaplan-Meier method, we found that the OS in high-risk cohorts was considerably shorter than OS in low-risk cohorts (Figure 2(f)). The ROC curve was used to assess the predictive power of TIS in CRC, and the time-dependent areas under the curve for TIS in predicting OS are 0.785 for 1 year, 0.806 for 3 years, and 0.832 for 5 years, respectively. (Figure 2(g)).

### 3.4. Clinicopathological Characteristics in Different Risk Score Groups

**Figure 3:**
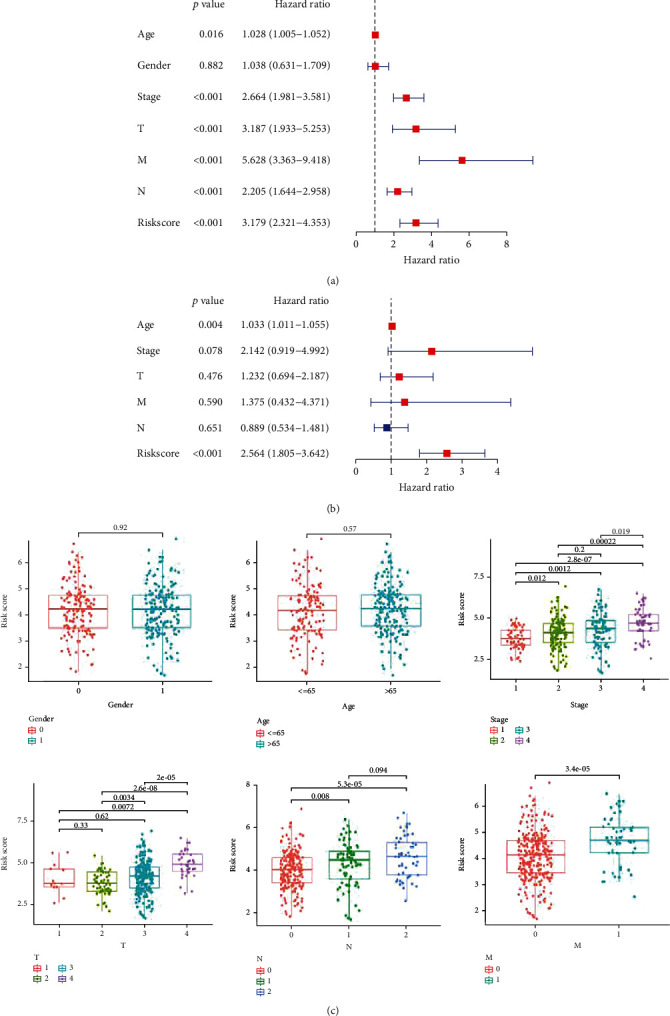
(a, b) Univariate and multivariate Cox regression analyses for OS in CRC. (c) Boxplots showing the stratification analysis of risk scores by clinical characteristics.

We further investigate the association between the TIS and overall survival. Univariate and multivariate Cox regression analyses indicated TIS was a variable independent of other clinical factors, including age, gender, stage, and TNM status (Figures 3(a) and 3(b)).

In addition, we compared the association of risk score and the clinicopathologic features in high- and low-risk groups. There was no significant difference in risk scores between different age groups (≤65 and >65) or gender (male and female). However, our results show that the tumor risk score of stages III-IV is much higher than that of stages I-II. Meanwhile, further analysis results show that CRC patients with late tumor stages and metastasis (lymph node or distant metastasis) exhibited higher risk scores (Figure 3(c)). The above results show that in these clinical subgroups, the established TIS prognostic model has a strong ability to predict the prognosis of CRC patients.

**Figure 4:**
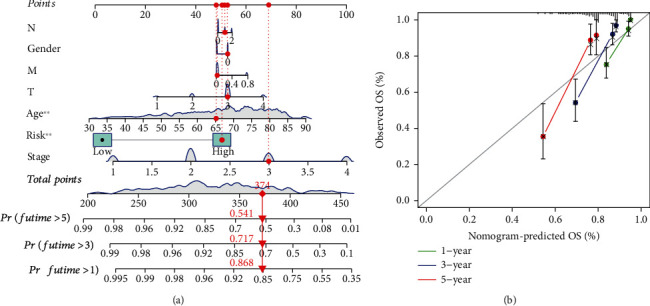
(a) Prognostic nomogram for predicting overall survival rate of CRC patients. (b) The 1-year, 3-year, and 5-year calibration curves of the nomogram model.

To provide a clinically appropriate method for predicting CRC patients' survival, we establish the nomogram plots to predict 1-, 3-, and 5-year survival rates for CRC patients based on TIS and clinicopathological prognostic factors (Figure 4(a)). The calibration curve shows that the predicted results of the model are in good agreement with the actual observation results (Figures 4(b)).

### 3.5. Analysis of Immune Status and Tumor Microenvironment

**Figure 5:**
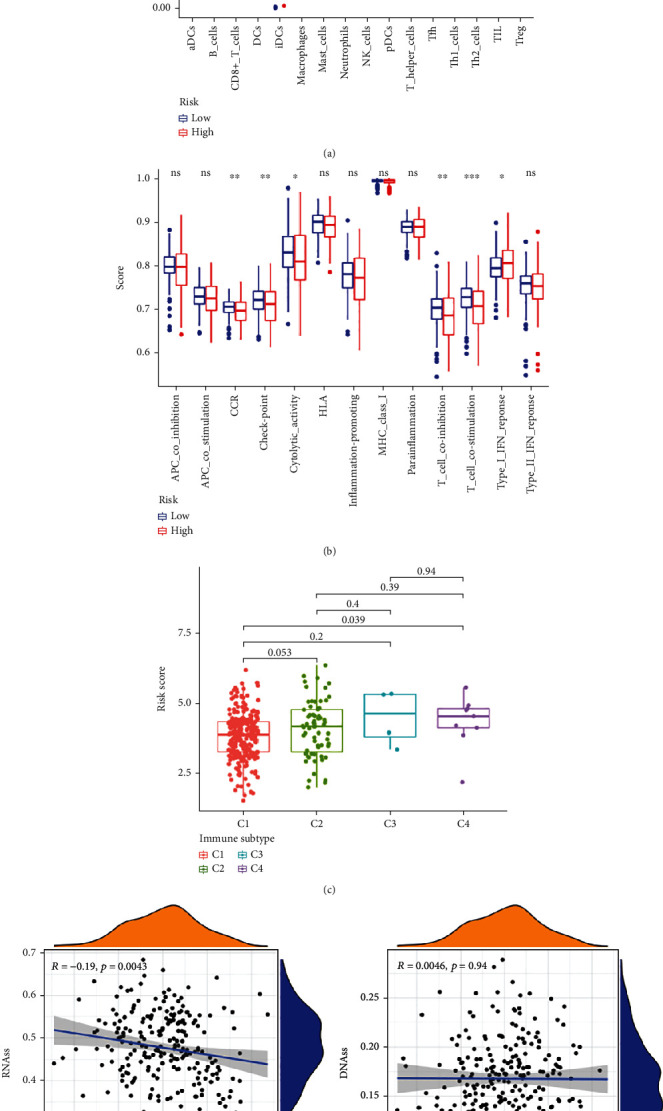
Association between TIS and Immune status. (a) Differences in characteristics of immune cells and (b) immune-related functions between high and low TIS scores. (c) Box plots comparing different immune infiltration subtypes and risk scores. (d) Scatter plot of correlation between risk score and RNAss and DNAss.

Immunity and tumor microenvironment play an important role in the process of tumorigenesis and malignant progression. To examine the correlation between TIS and immune status in CRC, single-sample GSEA (ssGSEA) enrichment analysis was performed. As shown in Figure 5(a), compared with the high-risk cohort, the scores of multiple immune cell types, such as CD8+ T cells, B cells, DCs, iDCs, pDCs, helper T cell 2 (Th2) cells, NK cells, Th1 cells, TILs, and Tregs, were higher in the low-risk cohort. Moreover, the scores of several pivotal immune-related functions, e.g., CCR, checkpoint, cytolytic activity, T cell coinhibition, T cell costimulation, and type I IFN response activity, were also significantly elevated in the low-risk group (Figure 5(b)).

Six types of immune characteristic modules were previously explored to describe immune states, including C1 (wound healing), C2 (INF-*γ* reaction), C3 (inflammatory), C4 (lymphocyte infiltration), C5 (immunologically quiet), and C6 (TGF-*β* response), we applied the ESTIMATE algorithm to determine the immune infiltrates of the selected four subtypes, and among the results, C4 had the highest risk score, while C1 possessed the lowest risk score (Figure 5(c)).

**Figure 6:**
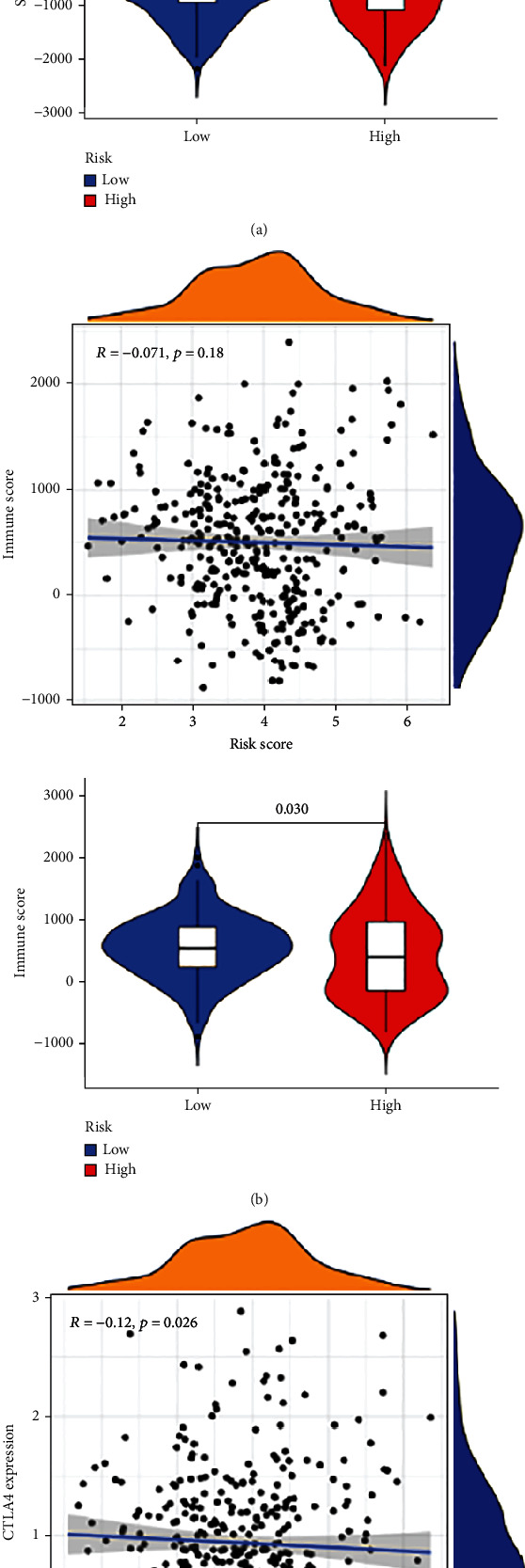
The correlation between risk score and (a) stromal score and (b) Immune Score. (c) Association between CTLA4 expression and risk score, and the expression difference of CTLA4 between different risk groups.

More and more evidence shows that the increased expression of tumor stemness (RNAss and DNAss) in tumor cells is highly correlated with drug resistance, cancer recurrence, and tumor proliferation. Therefore, we evaluated the correlation between DNAss and RNAss and risk score. Our study also compared the tumor immune microenvironment between the high- and low-risk score groups by using the stromal score and immune score. The results illustrated that the risk score was significant and negatively linked to RNAss (Figure 5(d)) but significantly positively linked to stromal score (*p* < 0.05) (Figure 6(a)). In addition, this study also found higher immune scores in the low-risk group (Figure 6(b)).

Immune checkpoint blockade (ICB) has demonstrated clinical success as a target for tumor immunotherapy. There are three main target sites of immune checkpoint therapy: PD-1, CTLA-4, PD-L1, and several other genes (e.g., LAG-3 and TIM-3). Therefore, the correlation between the risk score and the key gene of ICB was further investigated. The results showed that CTLA-4 was dramatically increased in patients with low-risk scores; correlation analysis further verified the above results (Figure 6(c)).

### 3.6. Pathway Analyses and Biological Function

**Figure 7:**
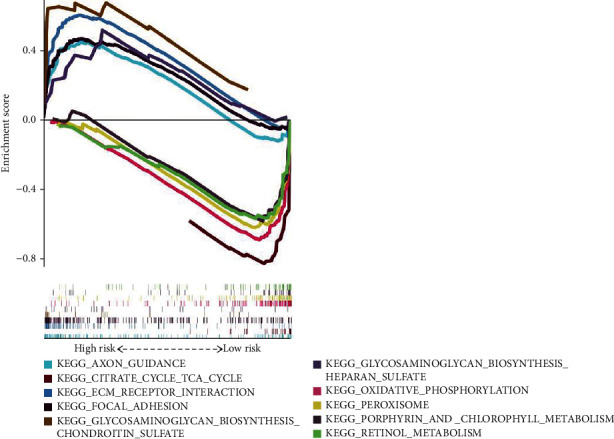
Exploration of biological signal pathways between the high or low TIS group in CRC by GSEA.

To compare the difference in molecular mechanisms involved in the low-risk and high-risk CRC patients, a GSEA was performed. In the high-risk score group, GSEA showed that many classical tumor-related pathways were enriched, including metabolic pathways and epithelial-to-mesenchymal transition (EMT) pathways. The significant 5 pathways enriched in the low-risk patients are closely correlated with immunity and metabolic pathways, which include the PEROXISOME pathway and the OXIDATIVE PHOSPHORYLATION pathway (Figure 7).

### 3.7. Future Potential Drug Targets for High-Risk Score Patients

**Figure 8:**
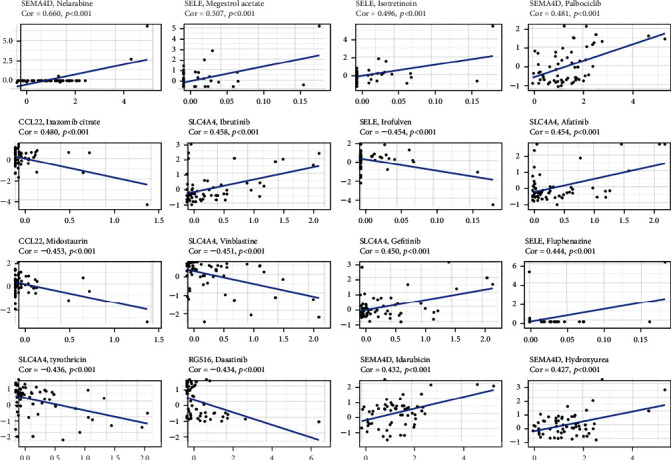
Association between prognostic gene expression levels and drug sensitivity.

To investigate the expression levels of prognostic TIS and measure the correlation between their expression levels and the sensitivity to FDA-approved anticancer drugs in the NCI-60 panel, the correlation analysis was performed, and the findings revealed that there were significant correlations between the IC50 values of many drugs and all prognostic genes. For instance, increased expression levels of SEMA4D, SELE, and SLC4A4 were linked to the higher sensitivity of cancer cells to a variety of chemotherapeutic drugs including nelarabine, megestrol acetate, isotretinoin, palbociclib, ibrutinib, etc. In addition, the high expression of CCL22 and RGS16 was related to greater drug resistance of cancer cells to midostaurin, ixazomib citrate, and dasatinib (Figure 8).

## 4. Discussion

CRC is a common digestive tract cancer associated with a high degree of morbidity and mortality, and the incidence of CRC has increased worldwide. Staging criteria were a commonly used method to guide the clinical outcomes, prognosis, and treatment of CRC; however, these are imperfect criteria due to the large variability of CRC patients. Here, we have demonstrated the independent prognostic value of TIS in CRC patients.

Nowadays, numerous studies show that systemic inflammation has a significant effect on the carcinogenic process, and it has become a new direction for cancer progression monitoring and therapeutic intervention [4]. Inflammatory response markers, containing serum albumin, C-reactive protein, and neutrophil to lymphocyte ratio, etc. [8–11], have been demonstrated with prognostic value in multiple cancers and have good predictive value in the prognostic evaluation of CRC [12, 13]. With the development of sequencing technology and bioinformatic approaches, TIS has been proved to predict the prognosis of renal carcinoma [14], pancreatic ductal adenocarcinoma [15], and lung cancer [16]. But the research on the correlation between TIS and the prognosis of patients with CRC is still limited. Understanding the role of TIS in CRC patients is valuable to improve the estimation of CRC prognosis and therapy decision-making.

In the present work, we designed to explore the expression pattern of inflammatory response-related genes in colorectal cancer and control tissues and screened DEGs. 80 DEGs were selected, and finally, 14 genes were selected to construct a TIS prognostic model. Using the median risk score, we divided the patients into the high-TIS-risk group and the low-TIS-risk group. Significantly different survival between high-TIS-risk and low-TIS-risk was verified, and CRC patients in the high-risk group had a worse prognosis. Furthermore, TIS was confirmed as an independent prognostic factor for CRC. The prognostic value of TIS was verified by the ROC curve analysis. The nomogram based on TIS and other clinical parameters showed high prediction performance and clinical decision-making value.

It is now becoming clear that the immune system is an indispensable participant in tumor occurrence, development, metastasis, and tumor treatment. As the fundamental innate immune response to perturbed tissue homeostasis, the relationship between TIS and immune signature in CRC needs further explored. Firstly, the relationship between immune status and risk score was evaluated by ssGSEA. The results revealed that the majority of immune cell subpopulations, cell functions, and signal paths were higher in the low-risk cohort, the infiltration degree of immune and stromal cells in CRC was evaluated by the ESTIMATE algorithm, and the results indicated that the immune scores were significantly lower in the high-risk group than those in the low-risk group. The above results show that the immunomodulatory effect of high-risk groups is inhibited, which may be the main reason for the poor prognosis of high-risk groups.

ICB has been shown to induce remarkable clinical success among various cancer types [17]. Interestingly, our study found that CTLA was differentially expressed in the high-risk and low-risk groups, but not PD1 or PDL1. The high-risk group was associated with a lower expression of CTLA-4 compared with the low-risk group, and the level of CTLA-4 expression showed a negative correlation with the risk score. Therefore, the TIS in CRC can help to predict the expression profile of immune checkpoint genes and may guide immunotherapy decisions.

Finally, research and analysis show that the increased expression of some prognostic genes was associated with increased drug sensitivity or resistance to a variety of FDA-authorized chemotherapeutic medicines. These findings confirm that these prognostic inflammatory response-related genes may serve as an intervention target for treatment to increase drug sensitivity or overcome drug resistance; this is also another key factor affecting the prognosis of CRC patients.

## 5. Conclusions

In conclusion, a novel predictive TIS of CRC was identified, and it was identified as an independent prognostic marker for patients with CRC. Through further prospective validation, TIS might play a vital role in the development of CRC and might be promising as a biomarker for CRC prognosis and treatment.

## Data Availability

The data used and analyzed in this article are available from https://portal.gdc.cancer.gov/.

## Supplementary Materials

Supplementary Materials Table S1: differentially expressed inflammation-related genes between the CRC and normal group.
