# Supplementary material for: Identification of a Novel Tumor Inflammation Signature for Risk Stratification, Prognosis Prediction, and Immune Status in Colorectal Cancer
Source: Biomed Res Int. 2022 Jul 16;2022:3465391. doi: 10.1155/2022/3465391 (PMC9308547; doi:10.1155/2022/3465391)
Supplement: Supplementary Materials — Table S1: differentially expressed inflammation-related genes between the CRC and normal group. [file 3465391.f1.docx]

Supplement Table1

Differentially expressed inflammation-related genes between the CRC and normal group

| gene | conMean | treatMean | logFC | pValue | fdr |
| --- | --- | --- | --- | --- | --- |
| ADRM1 | 38.94576 | 89.30647 | 1.197298 | 1.92E-22 | 3.25E-21 |
| AQP9 | 0.377835 | 1.564259 | 2.049651 | 2.55E-08 | 4.64E-08 |
| AXL | 11.2577 | 5.316734 | -1.0823 | 1.32E-13 | 4.10E-13 |
| BDKRB1 | 1.741562 | 0.615222 | -1.5012 | 2.87E-20 | 2.81E-19 |
| CCL20 | 15.01361 | 52.51273 | 1.806396 | 6.40E-08 | 1.12E-07 |
| CCL24 | 17.93748 | 62.08576 | 1.791285 | 2.31E-08 | 4.30E-08 |
| CCL5 | 30.63385 | 13.28125 | -1.20574 | 6.33E-12 | 1.76E-11 |
| CCR7 | 2.758948 | 1.242579 | -1.15078 | 2.82E-09 | 5.82E-09 |
| CD14 | 53.91229 | 26.2748 | -1.03693 | 1.22E-13 | 3.83E-13 |
| CD48 | 8.361301 | 2.573689 | -1.69989 | 3.84E-18 | 2.38E-17 |
| CD55 | 19.16081 | 40.05014 | 1.063648 | 1.74E-08 | 3.28E-08 |
| CD69 | 2.734842 | 1.240884 | -1.14009 | 7.65E-14 | 2.59E-13 |
| CD70 | 0.413089 | 1.136776 | 1.460424 | 0.000218 | 0.000291475 |
| CLEC5A | 0.070719 | 0.684562 | 3.275002 | 6.15E-18 | 3.46E-17 |
| CMKLR1 | 3.79334 | 1.404516 | -1.4334 | 2.16E-18 | 1.38E-17 |
| CSF3 | 0.360293 | 1.413084 | 1.971607 | 3.44E-08 | 6.16E-08 |
| CXCL11 | 1.57062 | 10.32532 | 2.71678 | 2.83E-15 | 1.22E-14 |
| CXCL6 | 0.312202 | 1.300568 | 2.058591 | 3.48E-09 | 7.12E-09 |
| EBI3 | 3.010972 | 1.269273 | -1.24623 | 5.58E-15 | 2.25E-14 |
| ADGRE1 | 0.498082 | 0.237147 | -1.0706 | 3.35E-10 | 7.42E-10 |
| EREG | 1.086556 | 12.07464 | 3.474145 | 1.26E-10 | 2.97E-10 |
| GNA15 | 2.379916 | 4.979796 | 1.065176 | 2.09E-07 | 3.47E-07 |
| GP1BA | 0.739301 | 0.349296 | -1.08171 | 1.78E-11 | 4.54E-11 |
| GPR183 | 8.830459 | 3.97724 | -1.15072 | 5.21E-12 | 1.47E-11 |
| IFITM1 | 83.12017 | 305.8652 | 1.879625 | 1.12E-18 | 7.43E-18 |
| IL10 | 0.580687 | 0.228178 | -1.3476 | 5.21E-14 | 1.83E-13 |
| IL10RA | 5.793857 | 2.233351 | -1.37531 | 1.15E-17 | 6.10E-17 |
| IL1A | 0.233505 | 1.320208 | 2.49924 | 6.06E-13 | 1.73E-12 |
| CXCL8 | 4.836498 | 41.10795 | 3.087383 | 8.80E-17 | 4.19E-16 |
| INHBA | 0.142835 | 5.184981 | 5.181916 | 7.31E-26 | 6.80E-24 |
| IRAK2 | 1.297766 | 4.523544 | 1.801423 | 8.71E-19 | 6.23E-18 |
| ITGB3 | 1.537549 | 0.535555 | -1.52153 | 2.83E-10 | 6.41E-10 |
| ITGB8 | 0.726993 | 1.644304 | 1.177463 | 4.02E-09 | 8.04E-09 |
| KCNA3 | 1.060131 | 0.233624 | -2.18198 | 6.78E-21 | 7.42E-20 |
| KCNMB2 | 0.131905 | 0.035348 | -1.89978 | 1.38E-13 | 4.20E-13 |
| LIF | 2.588631 | 9.425988 | 1.864454 | 9.70E-22 | 1.39E-20 |
| LPAR1 | 8.721036 | 1.965881 | -2.14932 | 1.12E-25 | 6.95E-24 |
| LY6E | 17.04587 | 67.97 | 1.995476 | 3.95E-15 | 1.63E-14 |
| MARCO | 6.536655 | 2.571963 | -1.34568 | 6.73E-05 | 9.70E-05 |
| MEP1A | 175.3738 | 38.96132 | -2.17032 | 4.16E-19 | 3.22E-18 |
| MET | 6.467036 | 26.93445 | 2.058276 | 7.05E-25 | 2.62E-23 |
| MMP14 | 29.2839 | 66.47375 | 1.182677 | 1.31E-11 | 3.43E-11 |
| MXD1 | 30.05831 | 10.29099 | -1.54638 | 3.86E-19 | 3.12E-18 |
| MYC | 26.30507 | 111.5899 | 2.084794 | 3.68E-24 | 8.55E-23 |
| NDP | 0.112929 | 0.444708 | 1.977448 | 0.000221 | 0.000294003 |
| NOD2 | 0.462522 | 1.370441 | 1.567046 | 1.50E-11 | 3.87E-11 |
| OLR1 | 0.152709 | 1.827176 | 3.58076 | 5.41E-18 | 3.24E-17 |
| OSM | 0.663382 | 2.635259 | 1.990033 | 6.98E-11 | 1.71E-10 |
| P2RX4 | 11.39716 | 4.979067 | -1.19473 | 4.37E-19 | 3.25E-18 |
| PCDH7 | 2.076789 | 0.773252 | -1.42534 | 1.97E-13 | 5.90E-13 |
| PDPN | 2.268441 | 7.222105 | 1.670718 | 2.80E-14 | 1.04E-13 |
| PIK3R5 | 1.392507 | 0.691402 | -1.01009 | 7.81E-14 | 2.59E-13 |
| PTGER4 | 17.25369 | 6.221828 | -1.47149 | 2.18E-23 | 4.51E-22 |
| PVR | 10.50334 | 23.43727 | 1.157956 | 3.86E-22 | 5.98E-21 |
| RGS16 | 1.821477 | 8.234198 | 2.17652 | 5.50E-21 | 7.10E-20 |
| RIPK2 | 3.0514 | 10.06964 | 1.722468 | 1.93E-25 | 8.97E-24 |
| SCN1B | 1.288798 | 0.540111 | -1.2547 | 7.07E-18 | 3.87E-17 |
| SELL | 4.511702 | 2.192705 | -1.04096 | 8.99E-08 | 1.53E-07 |
| SERPINE1 | 3.375118 | 14.57205 | 2.110194 | 9.35E-15 | 3.62E-14 |
| SLAMF1 | 1.211613 | 0.551772 | -1.13478 | 1.15E-13 | 3.70E-13 |
| SLC11A2 | 4.6326 | 11.2895 | 1.285088 | 6.45E-21 | 7.42E-20 |
| SLC1A2 | 0.056478 | 0.02792 | -1.01638 | 9.60E-09 | 1.84E-08 |
| SLC28A2 | 6.742137 | 1.72825 | -1.96389 | 1.07E-09 | 2.31E-09 |
| SLC31A2 | 0.98952 | 0.429779 | -1.20313 | 1.25E-17 | 6.45E-17 |
| SLC4A4 | 33.49025 | 1.422365 | -4.55738 | 6.49E-26 | 6.80E-24 |
| SLC7A1 | 9.359137 | 23.1607 | 1.307231 | 2.05E-20 | 2.12E-19 |
| SLC7A2 | 1.179082 | 0.555218 | -1.08654 | 8.24E-12 | 2.22E-11 |
| SPHK1 | 0.860883 | 3.143842 | 1.86864 | 3.03E-11 | 7.62E-11 |
| SRI | 111.4616 | 42.41125 | -1.39403 | 8.28E-20 | 7.33E-19 |
| STAB1 | 11.79719 | 4.006235 | -1.55812 | 9.87E-19 | 6.80E-18 |
| TACR1 | 0.363538 | 0.08545 | -2.08895 | 1.44E-15 | 6.55E-15 |
| TACR3 | 0.011692 | 0.003714 | -1.65434 | 4.25E-20 | 3.95E-19 |
| TIMP1 | 49.03874 | 199.5197 | 2.024537 | 1.02E-22 | 1.90E-21 |
| TLR1 | 1.493295 | 0.696564 | -1.10017 | 5.93E-14 | 2.04E-13 |
| TLR3 | 5.362538 | 1.431656 | -1.90523 | 2.80E-24 | 7.45E-23 |
| TNFAIP6 | 1.111619 | 2.638732 | 1.247182 | 9.16E-05 | 0.000131064 |
| TNFSF10 | 49.98954 | 20.0243 | -1.31987 | 2.18E-19 | 1.85E-18 |
| TNFSF15 | 0.854072 | 2.338318 | 1.453042 | 5.73E-21 | 7.10E-20 |
| TNFSF9 | 0.554725 | 6.077855 | 3.453719 | 5.89E-18 | 3.42E-17 |
| VIP | 33.80542 | 3.176065 | -3.41194 | 1.82E-24 | 5.66E-23 |
